# Economic burden of the therapeutic management of mental illnesses and its effect on household purchasing power

**DOI:** 10.1371/journal.pone.0202396

**Published:** 2018-09-10

**Authors:** Afis A. Agboola, Oluwaseun T. Esan, Oluwasegun T. Afolabi, Taiwo A. Soyinka, Adedunmola O. Oluwaranti, Adeniji Adetayo

**Affiliations:** 1 Child and Adolescent Mental Health Unit, Federal Neuropsychiatric Hospital Aro, Abeokuta, Ogun state, South west, Nigeria; 2 Department of Community Health, Faculty of Clinical Sciences, Obafemi Awolowo University, Ile-Ife, Osun State, South west, Nigeria; 3 Department of Research and Training, Research Unit, Federal Neuropsychiatric Hospital Aro, Abeokuta, Ogun state, South west, Nigeria; 4 Residency Training Program, Federal Neuropsychiatric Hospital Aro, Abeokuta, Ogun state, South west, Nigeria; University of Kentucky, UNITED STATES

## Abstract

Cost or burden of illness studies for mental illnesses has helped define the magnitude of their negative effects on the household, community and national economy. Despite its many benefits, there is a paucity of these studies among Nigerians being managed for mental illnesses. This study was aimed at assessing the economic burden of mental illnesses and its effect on household purchasing power. The study was descriptive cross-sectional in design conducted among 284 out-patients with five categories of mental illnesses at the Neuropsychiatric Hospital, Aro recruited via a systematic sampling technique. Data collection was quantitative using a semi-structured interviewer-administered tool. Participants provided the actual direct costs and estimates of indirect costs incurred during their most recent inpatient admission and their first clinic visit after discharge. Parametric and non-parametric statistical tests were conducted on the direct and estimated indirect costs respectively after testing them for normality using the Q-Q plot with statistical significance determined at p<0.05. Almost 96% of respondents finance their healthcare costs by themselves or their family with >50% earning <US$1.8 per day. Their mean direct and estimated indirect costs were (US$23.1±US$11.3 and US$15±US$28). There were no statistically significant differences in the mean direct and estimated indirect costs incurred by participants across the categories of mental illnesses. A significantly higher proportion of participants could afford the essential goods (88%) compared to those who could afford luxurious goods (29%) with p<0.001. The mean direct costs incurred by those who could afford the essential and luxurious goods were significantly higher than those who could not following a t-test. The median estimated indirect costs incurred by those who could not afford luxurious goods differed significantly from those who could with the Mann Whitney U-test. Participants with mental illnesses face a high economic burden in managing their condition with the majority unable to afford luxurious goods. Affordability was also associated with incurring more direct costs.

## Introduction

Cost of illness or burden of illness studies measure the prevalence of diseases, the effects on longevity and quality of life as well as the direct and indirect financial implications of the disease on individuals, their households, the community and national economy at large [1]. Assessing the economic burden of any illness, either a physical or mental disorder, involves estimating three types of costs which includes the direct cost, indirect cost and intangible cost[1]. Direct costs are those incurred to treat the illness and other supportive treatments including rehabilitation. Other direct costs include costs of transportation to and fro health facilities to receive care. Direct costs are often borne in large part by governments through social insurance schemes and to a lesser extent by private insurers if available. Indirect costs do not involve money spent on treatment but rather consists of lost productivity which is the value of what would have been produced in the absence of illness. Lost productivity could result from absenteeism or physical presence at work but with lower productivity because of ill health and in some cases, total withdrawals from the labour market owing to the chronic nature of the illness or premature death. Indirect costs are challenging to quantify but are critical for informing public policy and guiding discussion on what should be invested to prevent or treat the illness. Intangible costs include the pains, grief, fear of the unknown associated with managing a patient which are unquantifiable.

Mental health conditions are diseases of public health importance as they are said to constitute about 14% of the global burden of diseases [2]. They are one of the leading causes of workplace absenteeism or loss of job opportunity. In the United Kingdom, they are the second major causes of long-term occupational absence [3]. Illness typically leads to increased household spending on health services, and reduced time spent generating income. As a result of this, households may reduce their consumption of non-health goods, liquidate household savings or assets.

In Nigeria, provision of care for people with mental illness is grossly inadequate and supply of medication is not funded by the government. Primary care for mental health in Nigeria is at best nascent due to years of neglect, low manpower and perhaps worsened by the prevailing preference for spiritual or traditional care by people in the community[4, 5]. Modern health care services in this part of the world are concentrated in urban areas along with the healthcare professionals and facilities which further creates an imbalance of need and access [5]. Despite this, modern psychiatric hospitals are not readily patronized even by urban dwellers due to the stigma of mental illnesses and the high cost of care as the cost of treatment of the illness are usually paid for by affected individuals or their family[6, 7].

Treatment of mental illness could be long-term, especially those with major mental illness and it could be associated with high costs to the individual and the society. A primer, written by Segel on cost of illness studies [8] had described the overall magnitude of illness on individuals, groups and the national burden of illness in costs terms. The burden of illness estimates can be useful for establishing a population disease burden baseline against which future progress towards achieving disease prevention and health promotional goals may be measured. Cost or burden of illness studies particularly for mental illnesses can help define the magnitude of their negative effect on the household, community and national economy [9].

The lifetime prevalence of mental illness in a year in Nigeria according to Gureje et al in 2006 and the World Health Organization in 2007 was 12% [10, 11] though this was said to have been possibly underestimated [10]. It is also believed that the prevalence of mental illness is rising globally [12] however, developing countries appear less prepared to deal with the consequences in terms of cost and psychosocial impact. Apart from the lack of manpower and poor funding relative to the prevalence of mental illnesses, there has been a paucity of the cost of illness studies in literature among Nigerians being managed for mental illnesses, considering the known benefits of such studies. Very few studies in Nigeria have documented the cost of managing single selected mental disorders [13] and that was a long time ago, while a few others researched into the burden on caregivers of patients with mental illnesses [14, 15]. Gureje et al, studied the cost-effectiveness of a selected mental health intervention and not particularly the various types of costs incurred in the management of a mentally ill patient [6]. This study was aimed at assessing the economic burden of various categories of mental illnesses as well as the effect on patients’ household purchasing power. The specific objectives were to determine the direct and indirect costs of accessing treatment by patients’ diagnosed with mental illness, the effect of their monthly income on the direct and indirect costs incurred and the effect of the costs incurred on their household purchasing power.

## Methods

The study was a descriptive cross-sectional survey of out-patients on clinic visits at the Neuropsychiatric Hospital Aro, Abeokuta, South west, Nigeria. This tertiary health facility founded in 1954 is known as the foundation for providing community-based neuropsychiatric services. Participants were recruited at the outpatient clinic of this facility and a client exit interview conducted. Only participants aged 18 years and above who were recently discharged and were attending their first clinic visit after discharge were recruited. Other inclusion criteria included those who were either currently or previously engaged in some form of economic activities and not suffering from illnesses that can affect their sustained participation in the study.

Sample size determination was done using the Cochran formula for determining single proportions with a prevalence of psychiatric illness in Nigeria of 12% [11], and a 5% degree of precision at 99% confidence limit. This gave a minimum sample size of 281 which was rounded off to 300. The respondents were recruited via a systematic sampling technique with the expected number of patients expected at the clinic per day as the sampling frame. A simple random sampling through balloting was used to select the first respondent of the first ten clinic attendees per day, subsequently, every fifth patient who arrives for the clinic appointment was recruited if he or she meets the inclusion criteria. If not, he is replaced with the next arrival. If need be, a follow-up phone interview was conducted with respondents’ family members or caregivers for questions they could not provide answers to at the clinic.

Data collection was quantitative in nature and done using a structured interviewer-administered pre-tested questionnaire assessing variables such as socio-demographic characteristics, monthly income. Total direct costs incurred during their inpatient admission and first follow up clinic visits on drug purchase, investigations, hospital admission fees and transportation fees to and fro the facility within the last one year to the conduct of the study were obtained directly from the participants. This gives an impression of the cost of managing the illness during and immediately after an acute phase and the possible cost implications of managing it as a chronic illness with regular follow ups clinics.

The indirect costs were assessed by the respondents’ and their caregivers’ number of work-days absenteeism, the occurrence of loss of job, alternatives forgone or things the respondents’ household could no longer afford due to the illness. As a quantitative measure of the indirect costs, monetary estimates of these economic opportunities lost as a result of the illness were obtained. For example, respondents were asked how much they earned while working to give an estimate of the amount lost for not working due to the illness. Also, how much they usually earn from work per day, to give an estimate of the amount lost due to number of days they were absent from work. These monetary estimates of the indirect costs were provided by the participants and/or their caregivers who usually accompany them to the clinic.

Data analysis was done using the IBM SPSS (version 20.0). Test of normality using the Quantile-Quantile plot on the cost estimation data was done. Data on the monthly income of respondents and total direct cost incurred were found to be normally distributed while those of the indirect costs were not. The Student t-test was used to compare the means of the total direct costs across categorical data such as affordability of basic essential and luxurious commodities by respondents while the Mann Whitney U test was used to do same for the indirect costs. The One Way Analysis of Variance [16] test was used to compare means of the total direct costs across the various disease categories while the Kruskal Wallis test was used to do same for the indirect cost. Appropriate correlation tests were done to determine the correlation of respondents’ monthly income with the total and indirect costs incurred due to the illness. Level of statistical significance was set at p<0.05. Ethical approval was obtained from the Ethical review board committee of the Neuropsychiatric Hospital, Abeokuta, Aro Ogun State. A written informed consent was also obtained from respondents after due explanation of the study protocol.

## Results

Only 284 of 300 participants recruited fully participated in the study giving a response rate of 95%. There were more males than female respondents with a male to female ratio of 2.8:1. The mean age of respondents was (33.2 ± 8.8 years) Standard Deviation (SD) with the minimum and maximum ages of 18 and 65 years. Their median household monthly income was (₦16,915 (US$ 45.1) ± ₦22,000 (US$ 58.7) interquartile range (IQR) at the current rate of ₦375/USD.

A higher proportion of the respondents, (n = 143, 50%) were in the 30–45 years’ age bracket with 59% (n = 168) yet to be married. Majority of them were employed (n = 226, 80%), of which Artisan/Technician (n = 74, 26%) and trade/business (n = 72, 25%) predominates. Schizophrenia (n = 109, 38%), was the most common diagnosis among them followed by seizure disorders with co-morbid psychosis (n = 81, 29%). (Table 1)

**Table 1:** Socio-demographic characteristics of respondents.

Variables	Frequency (n = 284)	Percentage (%)
**Sex**		
Female	74	26.1
Male	210	73.9
**Age**		
18–29	119	41.8
30–45	143	50.4
46–65	22	7.8
**Religion**		
Christianity	189	66.5
Islam	95	33.5
**Marital status**		
Divorced/Separated	7	2.5
Married	106	37.3
Single	168	59.2
Widowed	3	1.1
**Educational Status**		
Primary	11	3.9
Junior secondary	13	4.6
Senior secondary	149	52.5
Tertiary/Postgraduate	111	39.1
**Occupation**		
Academician	14	4.9
Artisan/Technician	74	26.1
Civil servant	53	18.7
Non-governmental duty	10	3.5
Professionals	50	17.6
Students	6	2.1
Trade/Business	72	25.4
Uniform Officers	5	1.8
**Currently working**		
Yes	226	79.6
No	58	20.4
**ICD 10 Diagnosis**		
Bipolar Affective disorder (BAD)	41	14.4
Depression	40	14.1
Mental &Behavioural disorder (MBD)	13	4.6
Schizophrenia	109	38.4
Seizure disorder and acute morbid psychosis	81	28.5

The family was responsible for financing the treatment of a higher proportion of the respondents (n = 192, 68%) with only 4%, (n = 11) of them supported by their health insurance policy. The estimated total monthly household income for >50% of the respondents was ≤₦20,000 (≥US$ 53). Though, a higher proportion of the respondents (n = 249, 88%) could afford the essential basic needs such as food and clothing, a much less 29%, (n = 83) could acknowledge same for luxury goods such as cars. Also, less than a quarter of them, (n = 70, 25%) owned houses. (Table 2)

**Table 2:** Socio-economic characteristics of respondents and economic implications of the management of their mental illness.

Variables	Frequency (n = 284)	Percentage (%)
**Financier of respondent’s treatment**		
Self	81	28.5
Family member	192	67.6
Health Insurance / Government	11	3.9
**Total household income per month**		
≤ ₦20,000 (US$ 53.3)	167	58.8
> ₦20,000 to ≤₦50,000 (US$ 53.3- US$ 133.3)	82	28.9
>₦50,000 to ≤₦100,000 (US$ 133.3- US$ 266.7)	28	9.9
>₦100,000 (US$ 266.7)	7	2.5
**Can afford essential basic needs like food & clothing’s**		
Yes	249	87.7
No	35	12.3
**Can afford luxury goods like cars & other expensive commodities**		
Yes	83	29.2
No	201	70.8
**Respondent owns a built house by self**		
Yes	70	24.6
No	214	75.4
**Has ever been absent from work due to illness**		
Yes	134	47.2
No	150	52.8
**Days of Absence per month**		
0–3days	103	76.9
4–7days	22	16.4
>7days	9	6.7
**Has ever lost job due to the illness**		
Yes	58	20.4
No	226	79.6

Almost half of the respondents (n = 134, 47%) had been absent from work at some point due to the illness. For those who reported absenteeism from work due to the mental illness, the average number of days they were absent from work was (2.3 days ± 1.6 days S.D) with a higher proportion absent for less than three days. Unfortunately, almost a quarter of the respondents (n = 58, 20%) had actually lost their jobs due to the illness. (Table 2)

The minimum and maximum total direct costs incurred by respondents were ₦1,300 (US$ 3.5) and ₦28,000 (US$ 74.7) respectively. The mean estimated total direct cost of treatment was (₦8,645.40 (US$ 23.1) ± ₦4, 237.70 (US$ 11.3) S.D). The mean total direct costs incurred as well as the various components that made up the total direct costs were compared across the various categories of mental illnesses diagnosed. The mean direct costs on drugs were the highest of the component costs that made up the direct cost, across the various diseases categories. There were no statistically significant differences in the mean total direct costs nor any of its component costs within and across the various categories of mental illnesses diagnosed. (Table 3)

**Table 3:** Total direct cost incurred by participants across different psychiatric diagnoses.

Dependent variables	Independent variables	N	Means (₦)	Standard Deviation (₦)	Test of Statistical significance [17]	P-values
Transportation Cost	Bipolar Affective Disorder	41	**₦**2125.6 ($5.7)	**₦**1771.2 ($4.7)	F-test = 0.36 (4, 279)	p = 0.835
	Depression	40	**₦**2042.5 ($5.4)	**₦**1299.1 ($3.5)		
	Mental Behavioural Disorder	13	**₦**2584.6 ($6.9)	**₦**2516.9 ($6.7)		
	Schizophrenia	109	**₦**2003.1 ($5.3)	**₦**1610.1 ($4.3)		
	Seizure Disorder	81	**₦**2106.0 ($5.6)	**₦**1771.0 ($4.7)		
Laboratory Test Cost	Bipolar Affective Disorder	41	**₦**673.1 ($1.8)	**₦**1336.0 ($3.6)	F-test = 0.83 (4, 279)	p = 0.506
	Depression	40	**₦**860.0 ($2.3)	**₦**1024.0 ($2.7)		
	Mental Behavioural Disorder	13	**₦**542.3 ($1.4)	**₦**610.3 ($1.6)		
	Schizophrenia	109	**₦**873.9 ($2.3)	**₦**1374.6 ($3.7)		
	Seizure Disorder	81	**₦**1080.9 ($2.9)	**₦**1678.7 ($4.5)		
Drug Cost	Bipolar Affective Disorder	41	**₦**4478.0 ($11.9)	**₦**2872.0 ($7.7)	F-test = 1.34 (4, 279)	p = 0.256
	Depression	40	**₦**6232.5 ($16.6)	**₦**3042.1 ($8.1)		
	Mental Behavioural Disorder	13	**₦**4730.8 ($12.6)	**₦**2260.3 ($6.0)		
	Schizophrenia	109	**₦**5113.6 ($13.6)	**₦**3852.4 ($10.3)		
	Seizure Disorder	81	**₦**5046.0 ($13.5)	**₦**3497.9 ($9.3)		
Other Costs	Bipolar Affective Disorder	41	**₦**686.6 ($1.8)	**₦**2108.4 ($5.6)	F-test = 0.00 (4, 270)	p = 1.000
	Depression	38	**₦**584.2 ($1.6)	**₦**1592.6 ($4.2)		
	Mental Behavioural Disorder	13	**₦**0.0 ($0.0)	**₦**0.0 ($0.0)		
	Schizophrenia	109	**₦**518.3 ($1.4)	**₦**1587.4 ($4.2)		
	Seizure Disorder	77	**₦**551.3 ($1.5)	**₦**164,160.0 ($437.8)		
Total Direct Cost	Bipolar Affective Disorder	40	**₦**7887.5 ($21.0)	**₦**4133.4 ($11.0)	F-test = 1.10 (4, 278)	p = 0.356
	Depression	40	**₦**9690.0 ($25.8)	**₦**3887.5 ($10.4)		
	Mental Behavioural Disorder	13	**₦**7857.7 ($21.0)	**₦**3034.8 ($8.1)		
	Schizophrenia	109	**₦**8508.8 ($22.7)	**₦**4385.5 ($11.7)		
	Seizure Disorder	81	**₦**8816.5 ($23.5)	**₦**4392.0 ($11.7)		

Total direct cost consists of transportation cost per visit, laboratory investigation fees, drug cost and other cost including admission fees

The minimum and maximum estimated indirect costs were ₦1000 (US$ 2.7) and ₦90,000 (US$ 240) respectively. The mean estimated indirect costs were (₦5,626.80 (US$ 15) ± ₦10, 511.80 (US$ 28) S.D) while the median estimated indirect costs were (₦1500 (US$ 4) with ₦1500 (US$ 4) inter-quartile range). When the median estimated indirect costs across the various disease categories were ranked and compared, it was found not to be statistically significant. (Table 4)

**Table 4:** Indirect cost (INDC) estimated due to the illness across different psychiatric diagnoses.

Disease categories	N	Mean	Standard Deviation	Median	Mean rank	Test of statistical significance; p-value
Bipolar Affective Disorder	41	**₦**2526.8($6.7)	**₦**4533.0 ($12.1)	**₦**4000 ($10.7)	125.84	Kruskal Wallis: = 3.979, (df = 5);p-value = 0.409
Depression	40	**₦**2620 ($7.0)	**₦**3403.3 ($9.1)	**₦**2000 ($5.3)	148.11	
Mental and behavioural Disorder	13	**₦**5153.8 ($13.7)	**₦**7194.9 ($19.2)	**₦**2000 ($4.0)	173.35	
Schizophrenia	109	**₦**4686.5 ($12.5)	**₦**9843.1 ($26.2)	**₦**1500 ($4.0)	142.00	
Seizure Disorder	81	**₦**4802.6 ($12.8)	**₦**11995.1 ($32.0)	**₦**1500 ($4.0)	143.89	
Total	284	**₦**4138.2 ($11.0)	**₦**9237.7 ($24.6)	**₦**1500 ($4.0)		

INDC: Indirect cost consist of monetary value forgone by caregiver that accompany participants to the hospital; loss of monthly salary if the individual become unemployed due to the illness; or amount lost by the patient as a result of absent from work because of the illness

There were a higher proportion of respondents (n = 249, 88%) who could afford the basic essentials of life such as food and clothing. So also, there were a higher proportion who could not afford to have luxurious goods (n = 201, 71%). The difference in affordability of basic essentials goods and luxurious goods was statistically significant (χ2 = 199.411, degree of freedom (df) = 1; p<0.001) The mean total direct cost incurred by those who could afford the basic necessities of life was higher as well as for those who could afford to have luxurious goods was higher compared to those who could not. There were statistically significant differences in the mean total direct cost of those who could or could not afford the basic essentials of life (p = 0.024) and to have luxurious goods (p = 0.041)

However, the median estimated indirect costs incurred by respondents who could not afford to have the luxurious goods or not even the basic necessities of life were either higher or with a higher variability respectively compared with those who could afford these goods. This difference in their median estimated indirect costs was significant statistically only for affordability of the luxurious goods (p = 0.009) (Tables 5 and 6).

**Table 5:** Total direct costs incurred across respondents’ ability to afford basic essentials of life and luxurious goods.

Variables	N (%)	Mean	Standard Deviation	*Test of Significance*	*P-value*
*Afford Basic Essential commodities				t-test = -2.268; (df = 282)	p = 0.024
Yes	249 (88.0)	**₦**8849.5 ($23.6)	**₦**3341.1 ($8.9)		
No	35 (12.0	**₦**7128.9 ($19.0)	**₦**4308.2 ($11.5)		
**Afford Luxurious goods and services				t-test = -2.055; (df = 282)	p = 0.041
Yes	83 (29.2)	**₦**9436.1 ($25.2)	**₦**4588.6 ($12.2)		
No	201 (70.8)	**₦**8307.6 ($22.2)	**₦**4044.7 ($10.8)		

TDC: Total direct cost consists of transportation cost, laboratory cost, cost of medication and other hospital charges

*Basic essential commodities include food, clothes.

** Luxurious commodities include cars, air conditioning system, and refrigerator.

**Table 6:** Estimated indirect costs incurred across respondents’ ability to afford basic essentials of life and luxurious goods.

Variables	N (%)	Median	(Q_1_-Q_3_)	*Test of Significance*	*P-value*
*Afford Basic Essential Commodities				*U-test = 4302	p = 0.9
**Yes**	249 (88.0)	1500 ($4.0)	2,850–0.0 ($7.6- $0.0)		
**No**	35 (12.0)	0.0 ($0.0)	10,000–0.0 ($26.7- $0.0)		
**Afford Luxurious goods and services				*U-test = 6242	p = 0.001
Yes	83 (29.2)	0.0 ($0.0)	2000–0.0 ($5.3 - $0.0)		
No	201 (70.8)	2000 ($5.3)	4100–0.0 ($10.9- $0.0)		

INDC: Indirect cost consist of monetary value forgone by caregiver that accompany participants to the hospital, loss of monthly salary by the unemployed patient due to the illness and also amount lost by the patient due to absent from work because of hospital visit.

*Basic essential commodities include food, clothes.

** Luxurious commodities include cars, air conditioning system, and refrigerator.

Respondents’ monthly income as an independent variable was correlated with their total direct and estimated indirect costs incurred as the dependent variables. Findings showed that for the total direct cost, there was a mild to moderate positive correlation of respondents’ monthly income on the total direct cost for all the participants and participants in all the disease categories. This finding was statistically significant except for participants with seizure disorders only.

However, for the estimated indirect costs, there was a very mild positive correlation between it and respondents’ monthly income only for all the participants and participants being managed for Bipolar Affective Disorder (BAD); Depression and seizure Disorders. For participants with Mental and Behavioural Disorders (MBD) and Schizophrenia, a mild negative correlation with monthly income were observed. None but one of the findings correlating estimated direct cost with respondents’ monthly income was statistically significant except for the mild positive correlation seen with participants with Bipolar Affective Disorder (BAD), p = 0.017. (Table 7)

**Table 7:** Correlation of respondents’ monthly income on their total direct and estimated indirect costs of treating mental illnesses.

Cost variables	All participants (n = 284)		BAD (n = 37)		Depression (n = 40)		MBD (n = 13)		Schizophrenia (n = 109)		Seizure disorder (n = 81)	
	r	p-value	R	p-value	R	p-value	R	p-value	r	p-value	r	p-value
TDC**	0.249	<0.001	0.461	0.002	0.332	0.036	0.658	0.014	0.225	0.008	0.172	0.125
INDC*	0.016	0.712	0.295	0.017	0.078	0.516	-0.07	0.752	-0.085	0.237	0.025	0.765

TDC: Total direct cost consists of transportation cost, laboratory cost, cost of medication and other hospital charges. INDC: Indirect cost consist of monetary value forgone by caregiver that accompany patient to the hospital, loss of monthly salary by the unemployed patient due to the illness and also amount lost by the patient as a result of absent from work because of hospital visit.

** Pearson’s Correlation was done to compare respondents’ monthly income on the Total Direct costs.

*Kendal-tau Correlation was done to compare respondents’ monthly income on estimated In-direct cost.

## Discussion

From an economic point of view, it is particularly important to focus on morbidity among people of the working age, since this has such great impact on the economy and thus on public finances. In this study, all the respondents are in the working population. Though this was not intended to be so, rather it shows the importance of the high burden of mental illnesses among the working population and the negative consequences of these. The direct costs and estimated indirect costs incurred and provided by these participants were assessed across the different categories of mental illnesses these participants were being managed for at the study population. The effect of these costs on participants’ affordability of basic essential and luxurious goods and how these costs correlated with participants’ monthly income were also assessed in this study.

It is important to note that schizophrenia and other severe mental illnesses were more represented in the study, accounting for about 70% of cases, however this is similar to other studies conducted in referral Centres in Nigeria [18] and other developing countries [19, 20]. This study confirmed that most severe cases of mental illnesses are referred to tertiary centres for treatment.

Job losses and low productivity are often consequences of mental illness, however, the focus of this study was on persons with current or recent engagement in some form of economic activities and who were not suffering from acute illnesses that could affect their sustained participation in the study. This might explain the high employment rate of 80% observed in this study compared to the 67% currently employed and 61% engaged in economic activities among clients attending outpatient clinics of the same hospital as reported by Adelufosi and Mosanya respectively [21, 22]. In addition, higher rates of employment in patients with schizophrenia and other severe mental illness have been reported in developing countries as lack of state welfare provision probably necessitates the need to earn a living among psychiatric patients that are mentally stable [23].

Despite the fact that majority of the participants were working, yet a high proportion of them depended on their relatives to finance their treatment. This is not surprising as their average monthly income of US$ 45 is actually US$ 1.5 per day which is below the World Bank defined poverty line of US$ 1.9 and grossly inadequate to survive on. More than half of the respondents (n = 167, 59%) actually live on ≤ US$ 53 per month (≤US$ 1.8 per day). Therefore, a high proportion of patients with mental health disorders have poverty issues to grapple with. Indeed, they are at higher risk of extreme poverty as they are also prone to work absenteeism and actual loss of their jobs as seen from this study due to their impaired mental state which further worsens their poverty situation.

With the mean total direct cost of US$ 23.1 as the cost of treatment incurred for managing an acute phase of the illness and a one-time follow-up clinic visit, through Out of Pocket payment, makes the respondents prone to catastrophic health spending. Catastrophic health spending as defined by Xu et al is said to have occurred if a household’s financial contributions to the health system exceed 40% of income remaining after subsistence needs have been met in a year [24]. Unfortunately, only 4%, (n = 11) of them had any form of financial support from government or their employers such as a health insurance package to mitigate the effect of the catastrophic health spending on their household.

It could be feared that at least 12% (n = 35) of the respondents were already experiencing catastrophic health spending as they were no longer able to afford essential basic goods like clothing, food, and shelter as a result of payment for treatment of mental illness. Likewise, the majority of the respondents (n = 201, 71%) could not afford luxury goods like personal cars and houses. This inability of respondents to afford the basic necessities of life is an indication of poverty among them. The effect of poverty on mental health is said to occur largely in three ways. Firstly, the deprivation of basic necessities has a particularly strong impact on mental wellbeing and may be associated with psychological distress [25]. Secondly, unemployment or low-income earnings is an economic stressor and risk factor for mental illnesses [26]. Thirdly, people with serious mental disorders have high levels of unemployment rate or loss of job putting them at risk of poverty [27]. Evidence from the Western Balkans suggests that high levels of ‘out of pocket’ expenditures can increase the incidence of poverty and push households into poverty [28]. It is estimated that each year, 44 million households worldwide face catastrophic health expenditures [29]. This can also exert an overall negative impact on savings at the societal level.

At the household level, costs incurred in the acquisition of health services represent the resources that could have been used for other types of consumption had the disease or illness not occurred, taking into account the fact that the impact on the present value of future consumption depends partly on whether the costs are funded from current income, savings, sales of assets or borrowing. This is similar to what occurs in other low income countries where absolute monetary levels of household out of pockets expenditure on health generally or on a specific disease can be linked to household forgone spending for goods and services in order to relieve a relative measure of financial burden so as to take care of the present mental health challenges facing a member of the family [29]

The experience of patients regardless of their type of mental illness on the mean direct costs incurred and that of its various types was similar and appears to be on the high side. The costs of treating mental illnesses worldwide are exceedingly high. In 1995, patients diagnosed with depression had a higher annual health care cost and higher cost for every other category of care provided such as pharmacy and laboratory services than patients not diagnosed with depression [30]. In 1996, the total direct costs of mental health services in the United States was $69.0 billion, which was 7.3 percent of the total health spending for that year according to the United States Ministry of Health [31]. The cost of managing patients with mental illnesses have continued to be a major problem for the Canadian society. However, just as in other high-income countries, the Canadian government subsidies the treatment cost in addition to a robust health insurance policy [32]. In contrast to this, ‘‘out of pocket” payment for treatment by patients or their relatives is still very common in low-income countries like Nigeria.

This study also showed that the estimated indirect cost of mental illnesses was high, especially for those respondents who lost their job as a result of the illness and had to depend on family members for most things including hospital bills. The chronic nature of mental illness and in some severe cases, lack of independent living causes high estimated indirect cost as a result of lost productivity, abstentions from work and informal caregivers’ time spent on the patients. Indirect costs of mental illness resulting from lost productivity in the United States were estimated to exceed $78 billion [31]. In some high-income countries, a large portion of indirect costs are borne by employers but ultimately result in a lower gross domestic product and therefore amount to a loss for society [33]. Moreover, studies indicate that direct and indirect costs will increase in the coming decades and may become unsustainable if nothing is done to bring them under control [27]. One of the solutions experts proposed was more funding for mental health promotion, mental illness prevention, and early intervention. Such an investment would likely produce long-term savings to both the government and the people.

The mean total direct cost incurred by respondents who could not afford basic essentials of life nor the luxury goods were significantly lower than for those who could. This suggests that these respondents may not have been receiving adequate care as they should because of the barrier of lack of funds to cope with their expected treatment package. Likewise, the estimated indirect cost given by respondents unable to afford especially the luxurious goods was way higher significantly than that given by those who could. This also reiterates the high value these respondents place on their various alternatives forgone to manage their health conditions.

A mild to moderate statistically significant positive correlation was also found between respondents’ monthly income and their total direct health cost expenditure in this study. Thus, the higher or lower the income, the more the patients’ ability or not to afford to pay for health services when done “out of pocket”. This vertical inequality is one of the very essences of various interventions by government and international agencies towards reducing the effect of poverty or inability to pay on access to quality healthcare services. Unfortunately, many people including patients with mental health illnesses are yet to benefit from these government interventions like the National Social Health Insurance Schemes (NHIS). In Nigeria where just a fraction of people in the informal sector are covered in the scheme [34], unsurprisingly too, the majority of respondents in this study working in the informal sector were not covered in the scheme especially as they had not embraced the voluntary contributor program of the NHIS.

## Conclusions

Majority of the participants with mental illness studied live below the US$1.8 poverty line and therefore needed support, be it from the government or their family to cater for their treatment. Irrespective of the mental health condition diagnosed, participants incurred relatively high direct costs and perceived high indirect costs in managing their condition. It was easier for participants with higher monthly income earnings to procure the drugs, do their investigations and pay for all the necessary direct costs in managing their condition unlike those with poorer earnings and this implies inequity in accessing mental health services. Unaffordability of luxurious goods by participants’ households was more profound than affordability of basic essentials of life after incurring direct and indirect costs in the management of mental health conditions in Nigeria.

Findings from this study has shown that patient’s being managed for mental illness are exposed to high economic burden from managing the illness. This could be an additional stressor that may worsen their health status leading to more morbidity and mortality in them. Data collected on direct and indirect cost would have been more comprehensive if collected over a 1-year period to capture both the management of an acute phase and the chronic phase of the illness. However, the authors were able to obtain data for the acute phase and a one-time clinic appointment in the chronic phase of managing mental illnesses. It is recommended that a more standardized tool be used in determining cost estimates for indirect costs in further studies. Direct and Indirect costs incurred by patients on health insurance and compared with those not under an insurance cover. This may help substantiate the need to amend the act governing the National Health Insurance Scheme to make it compulsory and particularly for patients with mental illnesses.

## Supporting information

S1 File Questionnaire. (DOCX)
