# Supplementary material for: Economic burden of the therapeutic management of mental illnesses and its effect on household purchasing power
Source: PLoS One. 2018 Sep 10;13(9):e0202396. doi: 10.1371/journal.pone.0202396 (PMC6130881; doi:10.1371/journal.pone.0202396)
Supplement: S1 File — (DOCX) [file pone.0202396.s001.docx]

**APPENDIX**

SOCIO-DEMOGRAPHIC QUESTIONNAIRE

Dear respondent, this study is designed to elicit information on the economic burden and quality of life of people with mental illness in Neuropsychiatric Hospital Aro, Abeokuta. Your cooperation is needed to truthfully answer the questions below. All information will be strictly treated as confidential. The information will be used for academic purpose only. You have right to refuse participation in this study and it will not in any way interfere with your treatment in the hospital. Thanks.

Dr. Agboola A. A. Consultant Psychiatrist

08037048540.

Respondent consent.

I give my consent to voluntarily participate in the study after adequate information have been provided to me by the researcher.

Signature……………………………

SOCIO-DEMOGRAPHIC DATA

1. Sex: (a) Male [ ] (b) Female [ ]

2. Age: -------------------------------------

3. Religion: (a) Christian [ ] (b) Islam [ ] (c) Traditional [ ]

(d) Others (please specify):

4. Marital Status: (a) Single [ ] (b) Married [ ] (c) Divorced/Separated [ ] (d) Widowed [ ]

5. Educational Status: (a) No formal education [ ] (b) Primary [ ] (c) <JSS3 [ ]

(d) >SSS3 [ ] (e) Tertiary/Post-graduates [ ]

6. Ethnicity…………………

7. What is your occupation? …………………………………………………….

8. Are you currently working? Yes….. No…….

9. If currently working what is your monthly income/salary? ………….

10. If not currently working what was your last monthly income/ salary? …………….

HOUSEHOLD CHARACTERISTICS OF THE RESPONDENT (CAREGIVERS/RELATIVES TO BE INTERVIEWED IF NECESSARY)

11. How many people reside in your household?

12. What is the average monthly family income of your household: Please tick one

(a) <N20,000……..(b) <N50,000……(c) 50,000 to 100,000……(d) <N200,000……..(e) 200,000 to 500,000…….. (f) >5000, 000…….

13. What is the source of water in your household: (a) Tap in house [ ]

(b) Public tap [ ] (c) Tanker – truck or vendor [ ] (d) Well [ ] (e) Borehole [ ]

14. What types of sanitation facilities are available in your household? (a) Water closet [ ]

(b) Latrine [ ] (c) Open field [ ] (d) others, [ ] please specific ……………………………

15. Which type of house do you dwell in: (a) Duplex [ ] (b) Bungalow [ ] (c) Flats [ ] (d) A room [ ] (e) others ( ) please specific…………………………………

16. Are you the owner of the house? Yes [ ] No [ ]

MENTAL ILLNESS AND COST OF ACCESSING HEALTH SERVICES

17. ICD 10 diagnosis of the illness you are receiving treatment for in this hospital (Please check the patient case note) ………………………….

18. Is any members of your household have similar mental health problem Yes [ ] No [ ]

19. If yes, what is the relationship? ………………………………

20. When have you had this mental health challenges?

.......................................................................

21. Who is financing your treatment? A. Self ( ) B. Family member ( ) C. Health Insurance ( ) D. Government ( ) Other’s Please specify...................................

22. When did you start receiving treatment in this hospital?

23. Have you been admitted in hospital for mental illness in the past? Yes….. No……

24. a. If yes how many times have you been admitted in the hospital for mental illness?

b. What was the total amount spent while on your admission?

c. If more than one admission in the past please calculates the total amount spent on all the previous admission and writes the total and write the total…………………………………

d. When did you start attending this present outpatient clinic: Weeks……? Months…….. Year…………

25. How much are you currently spending on the treatment at outpatient clinic per month?

| TREATMENT | COST |
| --- | --- |
| Transportation |  |
| Testing e.g. Laboratory investigation and various tests |  |
| Drugs: Please tick as appropriate Typical drug …. Atypical drug…..  Anticonvulsant……. Others specify | Total cost spent on all the drugs every month = |
| Associated illness or any other expenditure in monetary terms |  |

26. Does this illness take you away from your place of work? Yes [ ] No [ ]

27. How many days/hours per week are you away from work due to the illness?

28. Does anybody follow you to the clinic………………………………………………...

29. If yes, who? (State relationship) ……………………………………………………….

30. Quantify in monetary term the cost spent or foregone by the person weekly or monthly when (s)he accompanied you to the hospital

31. Is there any activity you or your household member(s) performs before but could not do again due to this illness? please list them

32. What are the areas of financial difficulties in your household due to the cost of your treatment? OR what has change in your household as a result of payment for your treatment in the hospital?

Please tick as appropriate goods or services your household can afford again despite payment for your treatment in the hospital

1. My household can afford basic essential things like foods or clothing Yes….No….

2. My household can afford Luxury goods like cars and other expensive commodities. Yes… No….

Please list any other things you or your family cannot afford as a result of your treatment for this condition……………………………………………………………………………………………………………………………………………………………………………………………………………………………………………………………………………………

33. Have you lost your job since onset of your illness? Yes [ ] No [ ]

34. How long ago have you lost job?

35. Since you lost your job have you ever being engaged again in any other job but of lesser salary? Yes….. No…..

36. Has the treatment offered you so far improved your productivity at work? Yes…. No…….

37. Tick appropriately mental health professionals that has been involved in the care of your treatment (a) Psychiatrist (b) psychiatric Nurse (c) Psychologist (d) Social worker (e) Occupational therapist(f) others please specific……………………………..

| (B). WORK LIMITATIONS QUESTIONNAIRE (SELF-ADMINISTERED SHORT-FORM) Questions 1 through 5 ask about how your health has affected you at work during the past 2 weeks. Please answer these questions even if you missed some workdays.  Mark the “Does not apply to my job” box only if the question describes something that is not part of your job.  If you have more than one job, report on your main job only. In the past 2 weeks, how much of the time did your physical health or emotional problems make it difficult for you to do the following? |
| --- |

(Mark one box on each line a. and b.)

|  | Difficult all of the time (100%) | Difficult most of the time | Difficult some of the time (about 50%) | Difficult a slight bit of the time | Difficult none of the time (0%) | Does not apply to my job |
| --- | --- | --- | --- | --- | --- | --- |
| a. get going easily at the beginning of the workday | 🞏1 | 🞏2 | 🞏3 | 🞏4 | 🞏5 | 🞏6 |
| b. start on your job as soon as you arrived at work | 🞏1 | 🞏2 | 🞏3 | 🞏4 | 🞏5 | 🞏6 |

| These questions ask you to rate the amount of time you were  able to handle certain parts of your job without difficulty. |
| --- |

2. a. In the past 2 weeks, how much of the time were you able to sit, stand, or stay in one position for longer than 15 minutes while working, without difficulty caused by physical health or emotional problems? (Mark one box.)

| Able all of the time (100%) | 🞏1 |
| --- | --- |
| Able most of the time | 🞏2 |
| Able some of the time (about 50%) | 🞏3 |
| Able a slight bit of the time | 🞏4 |
| Able none of the time (0%) | 🞏5 |
| Does not apply to my job | 🞏6 |

2b In the past 2 weeks, how much of the time were you able to repeat the same motions over and over again while working, without difficulty caused by physical health or emotional problems? (Mark one box.)

| Able all of the time (100%) | 🞏1 |
| --- | --- |
| Able most of the time | 🞏2 |
| Able some of the time (about 50%) | 🞏3 |
| Able a slight bit of the time | 🞏4 |
| Able none of the time (0%) | 🞏5 |
| Does not apply to my job | 🞏6 |

| This question asks about difficulties you may have had at work. |
| --- |

3. In the past 2 weeks, how much of the time did your physical health or emotional problems make it difficult for you to concentrate on your work?

(Mark one box.)

| Difficult all of the time (100%) | 🞏1 |
| --- | --- |
| Difficult most of the time | 🞏2 |
| Difficult some of the time (about 50%) | 🞏3 |
| Difficult a slight bit of the time | 🞏4 |
| Difficult none of the time (0%) | 🞏5 |
| Does not apply to my job | 🞏6 |

| The next question asks about difficulties in relation to the people you came in contact with while working. These may include employers, supervisors, coworkers, clients, customers, or the public. |
| --- |

In the past 2 weeks, how much of the time did your physical health or emotional problems make it difficult for you to speak with people in-person, in meetings or on the phone? (Mark one box.)

| Difficult all of the time (100%) | 🞏1 |
| --- | --- |
| Difficult most of the time | 🞏2 |
| Difficult some of the time (about 50%) | 🞏3 |
| Difficult a slight bit of the time | 🞏4 |
| Difficult none of the time (0%) | 🞏5 |
| Does not apply to my job | 🞏6 |

| These questions ask about how things went at work overall. |
| --- |

In the past 2 weeks, how much of the time did your physical health or emotional problems make it difficult for you to do the following?

(Mark one box on each line a. and b.)

|  | Difficult all of the time (100%) | Difficult most of the time | Difficult some of the time (about 50%) | Difficult a slight bit of the time | Difficult none of the time (0%) | Does not apply to my job |
| --- | --- | --- | --- | --- | --- | --- |
| a. handle the workload | 🞏1 | 🞏2 | 🞏3 | 🞏4 | 🞏5 | 🞏6 |
| b. finish work on time | 🞏1 | 🞏2 | 🞏3 | 🞏4 | 🞏5 | 🞏6 |
